# The Illinois State Medical Society

**Published:** 1851-05

**Authors:** 


### Part 4.—Editorial.

### ARTICLE I.

### ILLINOIS STATE MEDICAL SOCIETY.

We would put all of our readers that reside in Illinois, in mind of the approaching meeting of the State Medical Society, so that they may be prepared to attend it. The meeting will be held in the city of Peoria, on the first Tuesday in June next.

That our friends may be apprized of the qualifications for membership in the Society, we quote entire the clause of the constitution that refers to the subject.

"The members of this Society shall collectively represent and have cognizance of the common interests of the medical profession in the State of Illinois; and shall hold their appointment to membership, either as delegates from local institutions, as members by invitation, or as permanent members.

"*The Delegates* shall receive their appointment from permanently organized medical societies, medical colleges, hospitals, lunatic asylums, and other permanently organized institutions of good standing in the State of Illinois. Each delegate shall hold his appointment for one year, and until another is appointed to succeed him, and shall participate in all the business and affairs of the society.

"Each local society shall have the privilege of sending to the society one delegate for every five of its regular resident members, and one for every additional fraction of more than half of this number. The faculty of every regularly constituted medical college or chartered school of medicine, shall have the privilege of sending two delegates. The professional staff of every chartered or municipal hospital, and every other permanently organized medical institution of good standing shall have the privilege of sending one delegate.

"*The Members by Invitation* shall consist of practitioners of reputable standing from any part of the United States. They shall receive their appointment by invitation of the meeting, after an introduction from any of the members present, or from any of the absent permanent members. They shall hold their connection with the society until the close of the session at which they were received, and may participate in the discussions without the right of voting.

" *The Permanent Members* shall consist of all those who have served in the capacity of delegates, and of such other members as may receive the appointment by unanimous vote.

" Permanent members shall at all times be entitled to attend the meetings, and participate in the affairs of the society, so long as they shall continue to conform to its regulations, but without the right of voting; and when not in attendance, they shall be authorized to grant letters of introduction to reputable practioners of medicine residing in their vicinity, who may wish to participate in the meetings as provided for members by invitation.

"Every member elect, prior to the permanent organization of the annual meeting, or before voting on any question after the meeting has been organized, must sign these regulations, inscribing his name and address in full, specifying in what capacity he attends, and, if a delegate, the title of the institution from which he has received the appointment."

We hope there will be a general attendance of all those who were elected permanent members last year, as well as a full representation from the various societies and other institutions entitled to representation. From the interest we have heard expressed, we feel authorized to say that there will be a full delegation from Chicago.

